# Racial and Ethnic Differences in Physical Activity and Bone Density: National Health and Nutrition Examination Survey, 2007–2008

**DOI:** 10.5888/pcd10.130183

**Published:** 2013-12-26

**Authors:** Elizabeth Vásquez, Benjamin A. Shaw, Lenore Gensburg, Daniel Okorodudu, Leonor Corsino

**Affiliations:** Author Affiliations: Benjamin A. Shaw, Lenore Gensburg, University at Albany, SUNY, Rensselaer, New York; Daniel Okorodudu, Leonor Corsino, Duke University Medical Center, Durham, North Carolina.

## Abstract

**Introduction:**

Participation in regular physical activity (PA) may help maintain bone health as people age. However, most American adults do not engage in the recommended minimum levels of PA, and there are racial/ethnic differences in PA participation. This study aimed to determine whether current physical activity is related to bone density in a racially/ethnically diverse sample after controlling for age, sex, body mass index, poverty–income ratio, tobacco use, vitamin D and calcium intake, and use of osteoporosis medications.

**Methods:**

We obtained data on femoral bone mineral density for 2,819 adults aged 40 to 80 years who self-reported their race/ethnicity on the 2007–2008 National Health and Nutrition Examination Survey. Data on PA levels were obtained by self-report. We used linear regression models to examine the association between PA and bone density for each racial/ethnic group.

**Results:**

A greater percentage of non-Hispanic blacks (60.9%) and Hispanics (53.3%) reported low levels of PA than non-Hispanic whites (45.3%, *P* < .001). Non-Hispanic blacks (16.3%) and Hispanics (18.5%) had a lower prevalence of osteopenia than non-Hispanic whites (25.5%; *P* = .01) but were similar in the prevalence of normal and osteoporosis categories when compared with whites. There was a 0.031 g/cm^2^ difference in bone density between those in the high PA versus the low PA category (*P* = .003). This association remained (β = 0.027, *P* < .001) after adjusting for race/ethnicity, sex, body mass index, poverty–income ratio, tobacco use, and use of osteoporosis medications.

**Conclusion:**

Despite lower levels of activity, blacks and Hispanics were not more likely to have osteoporosis, and high levels of activity were significantly associated with higher bone density even when controlling for race/ethnicity and confounders. The lack of consistency in bone density differences suggests that the cause of the differences maybe multifactorial.

## Introduction

Osteoporosis is a chronic condition that is a major public health problem in the United States and around the world primarily because of the increased morbidity and mortality associated with osteoporotic fractures (1,2). Osteoporosis disproportionately affects the elderly, reducing their quality of life and contributing to declines in functioning, which in some instances can lead to the inability to remain in the community (2,3). In the past decade, large epidemiologic studies, using mostly non-Hispanic white samples, have contributed to understanding the risk factors of patients with osteoporosis and those who fracture (2,4). These studies suggest that men, women, and racial/ethnic minorities differ in terms of risk factors for osteoporosis and osteoporosis-related fractures (1,5). Furthermore, most research indicates that physical activity (PA) may help maintain bone health as people age (6). However, most American adults (54.6%) do not engage in the recommended minimum levels of PA (7), and there are racial/ethnic differences in PA participation (7,8). With the expected increase in the US minority population (38.9% in 2012 to 54.0% by 2050) (9,10), the public health importance of understanding racial and ethnic differences in PA and their relation to bone mineral density becomes more relevant.

Differences in the associations between PA and the prevention of bone loss and related fractures by race/ethnicity have been evaluated with conflicting results (11–13). We aimed to determine whether current physical activity is related to bone density in a racial/ethnically diverse sample of adults aged 40 to 80 years, after controlling for age, sex, body mass index (BMI), poverty–income ratio, tobacco use, vitamin D and calcium intake, and current osteoporosis treatment.

## Methods

We conducted this cross-sectional study using data from the 2007–2008 National Health and Nutrition Examination Survey (NHANES) (14). NHANES uses a stratified, multistage probability design to select a nationally representative sample of all races/ethnicities. NHANES collects data via household interviews and a physical examination, which includes a bone mineral density test conducted in a mobile examination center. Full details on survey methods and physical examination as well as bone mineral density measurement protocol are published elsewhere (15). The sample (n = 2,819) was restricted to men and women aged 40 to 80 years who participated in the mobile examination component and who completed the bone mineral density analysis by dual-energy X-ray absorptiometry (DXA) scan in NHANES 2007–2008, the most recent administration for which data are available. Because we used de-identified data, institutional review board approval was not required for the study.

DXA is the gold standard for assessing bone mineral content and bone mineral density (16). The left hip has been proposed as the reference skeletal site for defining osteoporosis. Therefore, bone mineral density of participants was measured at the left hip in pencil-beam mode by DXA (QDR 1000; Hologic; Waltham, Massachusetts) at the mobile examination center (16). Prevalence estimates of normal bone density, osteopenia, and osteoporosis, derived on the basis of bone mineral density, were calculated by using World Health Organization diagnostic criteria (16).

Physical activity was assessed by self-report. The PA questionnaire ascertained information on low-, moderate-, and vigorous-intensity PA over the past 30 days (17). Survey participants were asked to review hand cards that listed examples of low-, moderate-, and vigorous-intensity PA. The metabolic equivalent, a ratio of a person’s working metabolic rate relative to the resting metabolic rate, was used for the calculation of a categorical indicator: total time spent in PA during a typical week. The number of days, as well as the intensity of the PA, was taken into account. The 3 levels of PA used in this study are low, moderate, and high, based on metabolic equivalents scores and calculated according to the Global Physical Activity Questionnaire Analysis Guide (17).

### Covariates

The variables known to affect bone density included in the analysis were race/ethnicity, age, family poverty–income ratio, education level, BMI index, calcium and vitamin D intake, tobacco use, and use of osteoporosis medications. Race and ethnicity were assessed by self-report, and participants were categorized in mutually exclusive categories as non-Hispanic white, non-Hispanic black, Hispanic, and other. Data on age were collected by self-report and modeled continuously in years.

Poverty–income ratio is the ratio of income to the family’s poverty threshold as determined by family size and composition. Poverty–income ratio was used as a measure of socioeconomic status; values of less than 1.00 represent a family living below the official poverty threshold, whereas values at or above 1.00 indicate income at or above the poverty threshold. Poverty–income ratio was then categorized for analyses (<2, 2–5 and >5) to reflect living wage standards (18).

Self-reported education was categorized as less than high school education, high school diploma or general educational development, and more than high school. BMI (calculated as weight in kilograms divided by the square of height in meters, kg/m^2^) was available from the household and mobile examination center interviews and was categorized using Centers for Disease Control and Prevention criteria (18.5–24.9, normal; 25.0–29.9, overweight; and ≥30, obese) (19). 

Information on calcium and vitamin D intake was collected by a 24-hour dietary recall food frequency questionnaire as part of the in-person household interview and at the health examination in a mobile examination center. A second 24-hour dietary recall questionnaire was administered approximately 10 days later. Both 24-hour recalls were collected by using the US Department of Agriculture’s automated multiple-pass method (20). Information on use of dietary supplements included the participant's use of vitamins, minerals, herbs, and other dietary supplements over the past 30 days. The average daily intake of calcium and vitamin D from dietary supplements was calculated for participants according to the reported number of days supplements were used, the reported amount taken per day, and the serving size units recorded from the product. Calcium obtained from antacids was included. For this analysis, calcium was standardized to the elemental form in milligrams, and vitamin D was standardized to the microgram metric for comparison to the Dietary Reference Intake recommendations. Tobacco use was determined by self-report and categorized as never smoked, past smoker, and current smoker. Information on use of osteoporosis medications (yes or no) was also collected by self-report.

### Analysis

We calculated descriptive statistics as weighted means and percentages to describe the demographic characteristics of the sample and used bivariate analysis to evaluate the relationship between categorical PA and bone density differences, tested using χ^2^ tests for proportions as appropriate. We tested differences by race/ethnicity and sex in the association between PA and bone mineral density for significance by including first-order interaction terms (eg, physical activity × race/ethnicity and physical activity × sex) in linear regression models. We calculated bone mineral density distribution by race/ethnicity and level of PA in the total sample. The interactions testing racial/ethnic or sex differences in the relationship between PA and bone mineral density were not significant at *P* < .05, so we opted not to include the interaction terms in the final models. We used linear regression analysis when appropriate to assess the potential effect of PA on bone mineral density, after adjusting for age, family poverty–income ratio, education, BMI, calcium and vitamin D intake, tobacco use, and use of osteoporosis medications.

For all analyses we used the appropriate sample weights, taking into consideration the unequal probabilities for selection as described in the NHANES website (www.cdc.gov/nchs/surveys.htm). Statistical analysis was conducted using SAS version 9.2 (SAS Institute, Inc, Cary, North Carolina). A *P* value less than .05 was considered significant.

## Results

The mean age of the sample was 51 years, and 48.1% were men (Table 1); 50.8% of participants were non-Hispanic white, 20.8% were non-Hispanic black, and 28.4% were Hispanic. Among non-Hispanic white participants, 13.6% had less than a high school education; the proportion with less than a high school education was higher among non-Hispanic blacks (30.6%) and Hispanics (46.6%). Approximately 73% of the sample had bone mineral density values within the normal range (T score standard deviation [SD], −1 to 1, of the young adult mean). Of participants who had osteopenia, 25.5% were non-Hispanic white, 16.3% were non-Hispanic black, and 18.5% were Hispanic (T score SD, −1 to −2.5). Approximately 3% of the total sample had bone mineral density values within the osteoporosis range (SD, −2.5 or lower). In addition, 33.9% of non-Hispanic whites, 41.9% of non-Hispanic blacks, and 39.7% Hispanics were obese, and 5.2% of the total sample used medications for osteoporosis. Most participants did not meet the dietary recommendations for calcium and vitamin D intake (62.1% and 88.5%, respectively).

**Table 1:** Prevalence of Sociodemographic and Health-Related Characteristics, National Health and Nutrition Examination Survey, 2007–2008

Characteristic	Non-Hispanic White (n = 1,433)	Non-Hispanic Black (n = 585)	Hispanic (n = 801)	*P* Value
**Sex, %**				
Male	48.1	46.1	51.3	.30
Female	51.9	53.9	48.7	
**Aged 65–80 y, mean**	56.2	54.1	53.0	NA
**Education level, %**				
Less than high school	13.6	30.6	46.6	<.001
High school diploma or GED	26.3	22.4	19.6	
More than high school	60.1	47.0	33.8	
**Bone mineral density, %**				
Normal	71.6	81.5	78.3	.01
Osteopenia	25.5	16.3	18.5	
Osteoporosis	2.9	2.2	3.1	
**Body mass index, %**				
Normal (<25.0 kg/m^2^)	28.3	25.7	17.3	<.001
Overweight (25.0–29.9 kg/m^2^)	37.8	32.5	43.0	
Obese (≥30 kg/m^2^)	33.9	41.9	39.7	
**Physical activity level, %**				
Low	45.3	60.9	53.3	<.001
Moderate	21.2	14.0	17.1	
High	33.5	25.1	29.6	

Abbreviation: NA, not applicable; GED, general educational development.

The prevalence of low levels of PA was higher among non-Hispanic black men (52.2%) than among non-Hispanic white (39.3%) and Hispanic men (41.5%) (Table 2). Similarly, the prevalence of low levels of PA was higher among non-Hispanic black women (68.3%) than among Hispanic women (65.7%) and non-Hispanic white women (50.9%). Non-Hispanic white men (41.8%) and Hispanic men (41.5%) were more likely to participate in high levels of PA than non-Hispanic black men (32.7%). The PA distribution levels among women were similar; 25.8% of non-Hispanic white women reported high levels of PA whereas 18.6% of non-Hispanic black women and 16.8% of Hispanic women reported high levels.

**Table 2:** Sex Differences in Physical Activity, by Race/Ethnicity and Bone Mineral Density Categories, National Health and Nutrition Examination Survey, 2007–2008

Characteristic	Activity Level, %			*P* Value
	Low	Moderate	High	
**Men**				
Non-Hispanic black	52.2	15.1	32.7	<.001
Non-Hispanic white	39.3	18.9	41.8	
Hispanic	41.5	16.8	41.5	
**Bone mineral density**				
Normal	38.0	19.6	42.4	.005
Osteopenia	70.5	14.5	15.0	
Osteoporosis	46.8	26.5	26.7	
**Women**				
Non-Hispanic black	68.3	13.0	18.6	<.001
Non-Hispanic white	50.9	23.3	25.8	
Hispanic	65.7	17.5	16.8	
**Bone mineral density**				
Normal	52.4	22.5	25.1	.47
Osteopenia	55.0	21.7	23.3	
Osteoporosis	67.1	13.6	19.3	

 In all race/ethnicity and bone density groups, a low level of PA was more prevalent than moderate or high levels of PA (Figure). Although the prevalence of low levels of PA was lowest among non-Hispanic whites (45.3%), the prevalence of osteoporosis was highest in this group (35%), followed by Hispanics (28.7%) and non-Hispanic blacks (22.5%).

**Figure:**
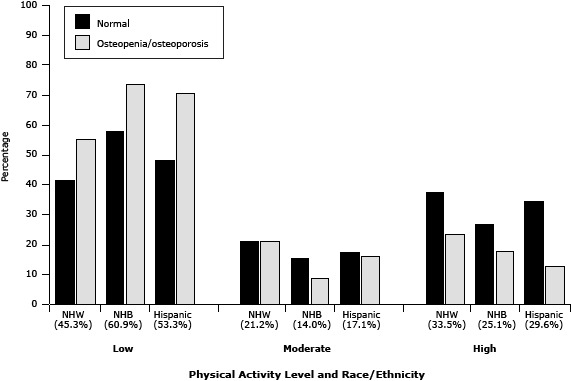
Prevalence of osteopenia/osteoporosis and normal bone mineral density among non-Hispanic whites, non-Hispanic blacks, and Hispanics, by levels of physical activity, National Health and Nutrition Examination Survey, 2007–2008. The percentage of participants in each category of PA is indicated in parentheses. Abbreviations: NHW, non-Hispanic white; NHB, non-Hispanic black. **Table:** 

Physical Activity Level and Race/Ethnicity	% of Participants in PA Category	Normal Bone Density, %	Osteopenia or Osteoporosis, %
**Low**			
Non-Hispanic white	45.3	41.4	55.2
Non-Hispanic black	60.9	57.9	73.7
Hispanic	53.3	48.5	70.7
**Moderate**			
Non-Hispanic white	21.2	21.2	21.2
Non-Hispanic black	14.0	15.2	8.7
Hispanic	17.1	17.4	16.3
**High**			
Non-Hispanic white	33.5	37.4	23.6
Non-Hispanic black	25.1	26.9	17.6
Hispanic	29.6	34.1	13.0

Because of the low proportion of participants who had osteoporosis, we assessed the association between PA and bone mineral density in this sample using linear regression models. In the full model (model 1), we found a 0.031 g/cm^2^ difference in bone density between those who had high levels of PA and those who had low levels of PA (*P* < .001) (Table 3). This association was attenuated in the final model (β = 0.027, *P* < .001) after adjusting for race/ethnicity, sex, BMI, poverty–income ratio, tobacco use, and use of osteoporosis medications (Table 3). Moreover, the results from model 1 and 2 show that non-Hispanic blacks had a higher bone mineral density than non-Hispanic whites (β = 0.070 for model 1 and 0.067 for model 2 and a *P* < .001 for both models), and both overweight and obesity were associated with bone mineral density (β = 0.068 and β = 0.134 respectively; *P* < .001) with normal bone mineral density as the reference group.

**Table 3:** Unadjusted and Adjusted Regression Models for the Association Between Physical Activity and Bone Density, National Health and Nutrition Examination Survey, 2007–2008

Characteristic	Model 1^a^		Model 2^b^	
	β	*P* Value	β	*P* Value
**Physical activity level**				
Low	1 [Reference]			
Moderate	0.016	.10	0.012	.13
High	0.031	<.001	0.027	<.001
**Race/ethnicity**				
Non-Hispanic white	1 [Reference]			
Non-Hispanic black	0.070	<.001	0.067	<.001
Hispanic	0.016	.09	0.014	.09
**Sex**				
Male	1 [Reference]			
Female	−0.107	<.001	−0.10	<.001
**Poverty index**				
More than living wage	1 [Reference]			
Living wage	−0.023	.13	−0.017	.10
Less than living wage	−0.044	<.001	−0.038	<.001
**Weight category**				
Normal (<25.0 kg/m^2^)	1 [Reference]			
Overweight (25.0–29.9 kg/m^2^)	0.066	<.001	0.068	<.001
Obese (≥30 kg/m^2^)	0.132	<.001	0.134	<.001
**Smoking status**				
Never	1 [Reference]			
Current	−0.016	<.001	−0.021	.01
Past	**−**0.010	.15	−0.007	.29
**Taking medications for osteoporosis**				
No	1 [Reference]			
Yes	−0.111	<.001	−0.100	.001
**Meets calcium intake dietary requirement**				
Yes	1 [Reference]			
No	NA		−0.002	.79
**Meets vitamin D intake dietary requirement**				
Yes	1 [Reference]			
No	NA		0.002	.81

Abbreviation: NA, not applicable.

a Full model adjusted for race/ethnicity, sex, family poverty–income ratio, body mass index, tobacco use, and use of osteoporosis medications.

b Final model adjusted for race/ethnicity, sex, family poverty–income ratio, body mass index, tobacco use, use of osteoporosis medications, and calcium and vitamin D intake.

## Discussion

We sought to determine whether current PA is related to bone mineral density in a racial/ethnically diverse sample of adults aged 40 to 80 after controlling for age, sex, BMI, poverty–income ratio, tobacco use, vitamin D and calcium intake, and use of osteoporosis medications. We found that participation in both high and moderate levels of PA was more prevalent among non-Hispanic whites than among non-Hispanic blacks and Hispanics. These findings are consistent with data showing that in a sample of adults aged 60 years or older, participants from minority groups reported lower levels of PA (21). Furthermore, our study showed that participants who engaged in low and moderate levels of PA had lower bone mineral density than those who engaged high levels of PA. A study published in 2001 indicated that women who engaged in low to moderate levels of PA had lower bone mineral density than those who engaged in high levels PA (22). However, our study also found that, among non-Hispanic blacks, rates of osteoporosis were low despite the low levels of PA. This paradoxical finding may suggest that either PA is not as strongly associated with bone mineral density among non-Hispanic blacks or that the high bone mineral density among non-Hispanic blacks is due to other factors not identified in this study.

In our study, a high bone mineral density was significant only for non-Hispanic blacks, which suggests multifactorial effects. Two studies examined the racial differences in bone mineral density in a diverse sample and similarly categorized participants as Hispanic, non-Hispanic white, and non-Hispanic black (23,24). In both studies, higher bone mineral density was found among non-Hispanic blacks than among non-Hispanic whites. However, neither of these studies included PA status.

Physical inactivity and poor nutrition are behavioral factors that increase BMI and perhaps adversely affect bone mineral density. These factors are also more prevalent in racial/ethnic minority and lower socioeconomic groups (25). For example, Hispanics and non-Hispanic blacks are reported to have higher BMI, consume less calcium and vitamin D, and engage in less PA than non-Hispanic whites (26,27). They are also reported to have lower levels of education, which can be associated with higher BMI, lower levels of income, and lower levels of fruit and vegetable consumption (7,28). Moreover, because poor bone health is reported to be enhanced by the cumulative effects of unhealthful behaviors occurring throughout life, it can be inferred that racial/ethnic minorities may be at increased risk for poor bone health as adults because of inadequate PA and nutrition during childhood, resulting in suboptimal achievement of peak bone mass earlier in life (29). Our findings also suggest that PA is not associated with bone mineral density as strongly among non-Hispanic blacks; other possible explanations for this association should be explored in future studies.

Our study has strengths and limitations. One strength was the use of a large community-dwelling sample representative of the US adult population. However, the NHANES data used in this study are subject to at least 2 limitations. First, a cross-sectional measurement of PA does not identify an individual’s true average activity over a given interval. Moreover, the type and length of PA participation plays a role in bone health; non-weight–bearing, high-force activity (eg, progressive resistance strength training) may benefit neck femur bone mineral density, which was the area measured in this study, but no other bone sites (12). This finding is important because PA declines with age while functional limitations, which impair the ability to participate in regular activity such as walking and climbing a flight of stairs, are also often reported to increase with advanced age. This decline in PA may help to explain the racial/ethnic group differences in PA and its effect on bone health.

In addition, self-reported PA data may include reporting errors because some respondents could not recall their past month’s level of PA or did not answer the question correctly. Furthermore, more participants reported high levels of PA than moderate levels, which may have resulted from self-report bias or misclassification. However, we calculated our categories using standard cutpoints provided by the measuring tool we used (17). Non-Hispanic blacks and Hispanics are more likely to report functional limitations and disability than non-Hispanic whites, which may explain the lower levels of PA among these 2 groups (30). Research also shows an increased prevalence of functional decline among those diagnosed with a chronic medical condition or conditions (eg, heart problems, fractures, arthritis, diabetes) than among those without these conditions (31), which were not measured in our study. Another potential limitation may be related to assessment of race/ethnicity. The Hispanic category includes whites, black Hispanics, and Mexican Americans, and we were not able to subclassify our Hispanic group into a more refined group of black and white Hispanics. Other limitations included the background confounders that were not evaluated, including menopause status, family history of osteoporosis or hip fracture, chronic diseases (eg, hyperparathyroidism, hypogonadism, malabsorption) and long-term use of medications (eg, steroids), predisposing participants to osteoporosis (1). To the extent that the associations studied here may change over time, these analysis should be replicated as more recent data becomes available. Despite these limitations, other studies corroborate our findings, showing that elderly minorities are disproportionally reported to reduce their ability to remain physically active, and this can be a contributing factor to the subsequent deterioration of bone health (2,28). However, the use of a validated PA scale that quantifies the degree of PA during the past month increases the reliability of our results.

The public health impact of inadequate levels of PA among older adults is significant, given the substantial increase in the percentage of racial/ethnic minority older adults expected in the next 40 years (23). With this increase, we need to gain a better understanding of the plausible explanations for the discrepancies in self-reported levels of PA among older adults and their effect on bone health data. However, additional research on the specific risk factors for osteoporosis among racial/ethnic minority populations continues to be limited, with lifestyle factors such as PA inconsistently reported to prevent bone loss and osteoporosis among minority populations (13,22).

This study concurs with previous literature showing that the prevalence of osteoporosis is higher among non-Hispanic whites despite their having higher levels of PA than their Hispanic and non-Hispanic black counterparts, that those with higher BMI are less likely to have osteoporosis, and that non-Hispanic blacks are least likely to have osteoporosis despite lower levels of PA. This study adds to the literature on the effect of PA in a racial/ethnically diverse sample of older adults, but longitudinal studies with larger numbers of racial/ethnic minorities are still needed to improve our understanding of these differences and to assess potential causes. Further longitudinal studies looking into the effect of PA and bone mineral density by race/ethnicity using a life-course approach are needed to better characterize the relationship between PA and bone mineral density.
